# The feasibility, safety and short-term clinical efficacy of laparoscopic anterior resection of rectal cancer with left colonic artery (LCA) preservation and natural orifice specimen extraction (NOSE)

**DOI:** 10.1186/s12893-022-01719-4

**Published:** 2022-08-10

**Authors:** Zhang Ke, Deng Zijian, Hu Hai, Yan Jin, Rui Yuanyi, Yi Bo, Zheng Yangchun

**Affiliations:** Department of Gastrointestinal Surgery, Sichuan Cancer Hospital, Chengdu, 610041 China

**Keywords:** Laparoscopic surgery, Rectal cancer, Natural orifice specimen extraction (NOSE), Left colon artery

## Abstract

**Background:**

Natural orifice specimen extraction surgery (NOSES) has the advantages of less postoperative pain, fast bowel function recovery, reduced hospital stay and better cosmetic effects. In our centre, anterior resection of rectal cancer with preservation of the left colonic artery (LCA) was performed using NOSES. The feasibility, safety and short-term clinical efficacy of the technique were discussed.

**Methods:**

A retrospective analysis was performed on 19 patients who underwent laparoscopic anterior resection of rectal cancer with left colonic artery preservation and natural orifice specimen extraction in the Gastrointestinal Surgery Center of Sichuan Cancer Hospital from September 2018 to December 2019. General information about the patients, perioperative data and short-term postoperative results were analysed.

**Results:**

All operations were completed smoothly, with an average operation duration of 304.36 ± 45.04 min, intraoperative bleeding of 76.31 ± 61.12 ml, first time off bed of 14.42 ± 3.56 h, first time to anus exhaust of 15.26 ± 8.92 h, first time to liquid diet of 2.94 ± 1.12 days, and average postoperative stay of 10.21 ± 3.13 days. Two patients developed temporary intestinal obstruction, and one patient developed pulmonary infection. All of them recovered well after active supportive treatment and were successfully discharged.

**Conclusion:**

Laparoscopic NOSES for rectal cancer with left colon artery preservation is safe and feasible, with satisfactory short-term results, and is worthy of further clinical investigation.

## Background

After more than 100 years of development, the surgical treatment of rectal cancer has experienced the evolution of the Miles operation, total mesorectal excision (TME), radical resection of rectal cancer with pelvic autonomic nerve preservation (PANP) and other important surgical methods and concept updates [1–3]. To date, the local recurrence rate, long-term survival rate and quality of life have been significantly improved. Currently, PANP radical (D3) resection based on the TME principle has become the standard surgical treatment for patients with rectal cancer. In the past 30 years, with the rapid development of minimally invasive technology and instruments, laparoscopic surgery has also been widely used in colorectal cancer surgery because of its advantages of minimal trauma, light pain and fast recovery [4–6]. However, traditional laparoscopic rectal cancer surgery requires an abdominal-assisted small incision to remove specimens and reconstruct the digestive tract. Postoperative complications such as incision infection, incision hernia and incision pain may occur, which not only affects the aesthetics but also reduces the quality of life [7–10]. The natural orifice specimen extraction surgery (NOSES), which has been developed in recent years, is called "minimally invasive surgery in minimally invasive surgery" because the specimen is removed through the natural orifice, such as the anus or vagina, without auxiliary incision in the abdomen to avoid the related complications caused by the incision. Our hospital has been performing laparoscopic colorectal cancer surgery with NOSE since 2016. Currently, more than 100 cases have been completed. On the basis of the previous stage, we performed NOSES with the left colorectal artery preserved in some rectal cancer patients. As of December 2019, 19 cases have been completed, and satisfactory results have been achieved. Now, the operation experience and recent curative effects are reported as follows.

## Methods

### General materials

The clinical data of patients who underwent laparoscopic rectal cancer NOSES with the left colonic artery preserved in the gastrointestinal surgery of Sichuan Cancer Hospital from September 2018 to December 2019 were collected retrospectively. There were 19 patients in this group, including 8 males and 11 females. The average age was 59.94 ± 10.52 years, the average body mass index was 23.15 ± 2.44 kg/m^2^, the average preoperative CEA level was 3.89 ± 5.62 ng/ml, the distance from the tumour to the anal margin was 5.55 ± 2.60 cm, and the length and diameter of the tumour was 3.89 ± 0.99 cm. There were 14 cases of AJCC stage II and 5 cases of AJCC stage III. All patients were confirmed to have adenocarcinoma by pathological biopsy before the operation, and 4 patients received neoadjuvant radiotherapy and chemotherapy before the operation.

### Surgical technique

The NOSE surgery for rectal cancer with the left colonic artery preserved follows the principles of D3 dissection, TME, and PANP. The pulling out method of surgical specimens is to select transanal pull-through for low rectal cancer (LAR-TAPT) or transanal specimen extraction for medium and high rectal cancer (HAR-TASE) according to the tumour location.

#### Body position and trocar position

The patient took the lithotomy position and lowered his right lower limb slightly to facilitate the free operation of the root of the inferior mesenteric artery (IMA). The pneumoperitoneum and operation channel were established by the five-hole method: a 10 mm trocar was placed on the umbilicus as the observation hole, a 12 mm trocar was placed at the right lower abdomen as the main operation hole, and a 5 trocar card was placed at the flat umbilicus of the right middle clavicle line, the flat umbilicus of the left middle clavicle line and the inner side of the left lower abdomen as the auxiliary operation hole.

#### Operation of IMA root

The mesentery was opened at the root of the sigmoid colon through an intermediate approach, and the left Toldt's space was entered. The approach to the IMA root is performed not too close to the root but from the head side of the IMA root to the bare area of the nerve in front of the abdominal aorta to better protect the superior hypogastric plexus (SHP) and more completely clean the lymph nodes of the blood vessel root.

#### Left colonic artery preservation

**Fig. 1:**
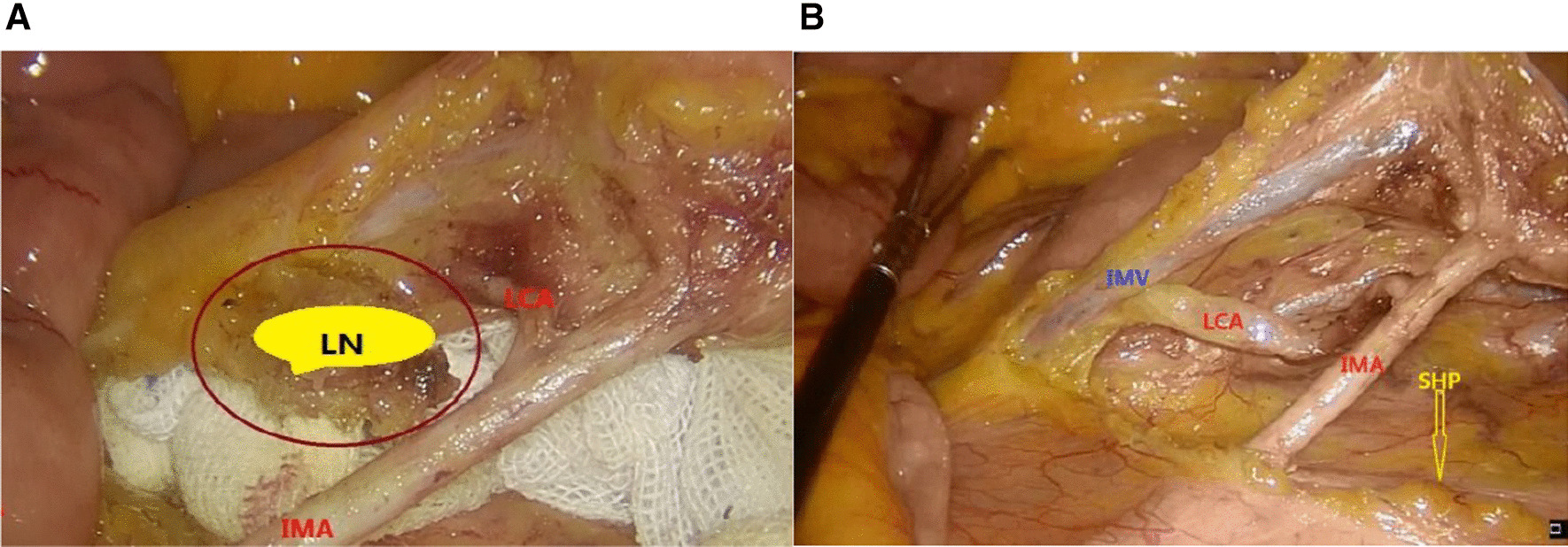
**A** D3 lymph node dissection at the root of the inferior mesenteric artery, **B** preservation of the left colonic artery; *LCA* left colonic artery; *IMA* inferior mesenteric artery; *SHP* superior hypogastric plexus

The key to the anatomical dissociation of the left colonic artery is to open the vascular sheath at the root of the IMA, sharply separate along the vascular sheath to the caudal side, and expose the left colonic artery and sigmoid artery. At the same time, the fat and lymphoid tissue between the inferior mesenteric artery and vein were cleaned. After exposing the inferior mesenteric vein (IMV), dissection was continued to the caudal side to expose the left colonic vein, and the IMA and IMV were cut off below the left colonic artery and vein, respectively (Fig. 1).

#### Dissection of mesorectum

According to the principle of TME, sharp dissection was performed to the caudal side along the avascular space between the visceral and parietal layers of the pelvic fascia, and the bilateral inferior hypogastric nerves and pelvic nerve plexus were protected until approximately 5 cm away from the distal end of the tumour or the levator ani plane.

#### Specimen extraction

**Fig. 2:**
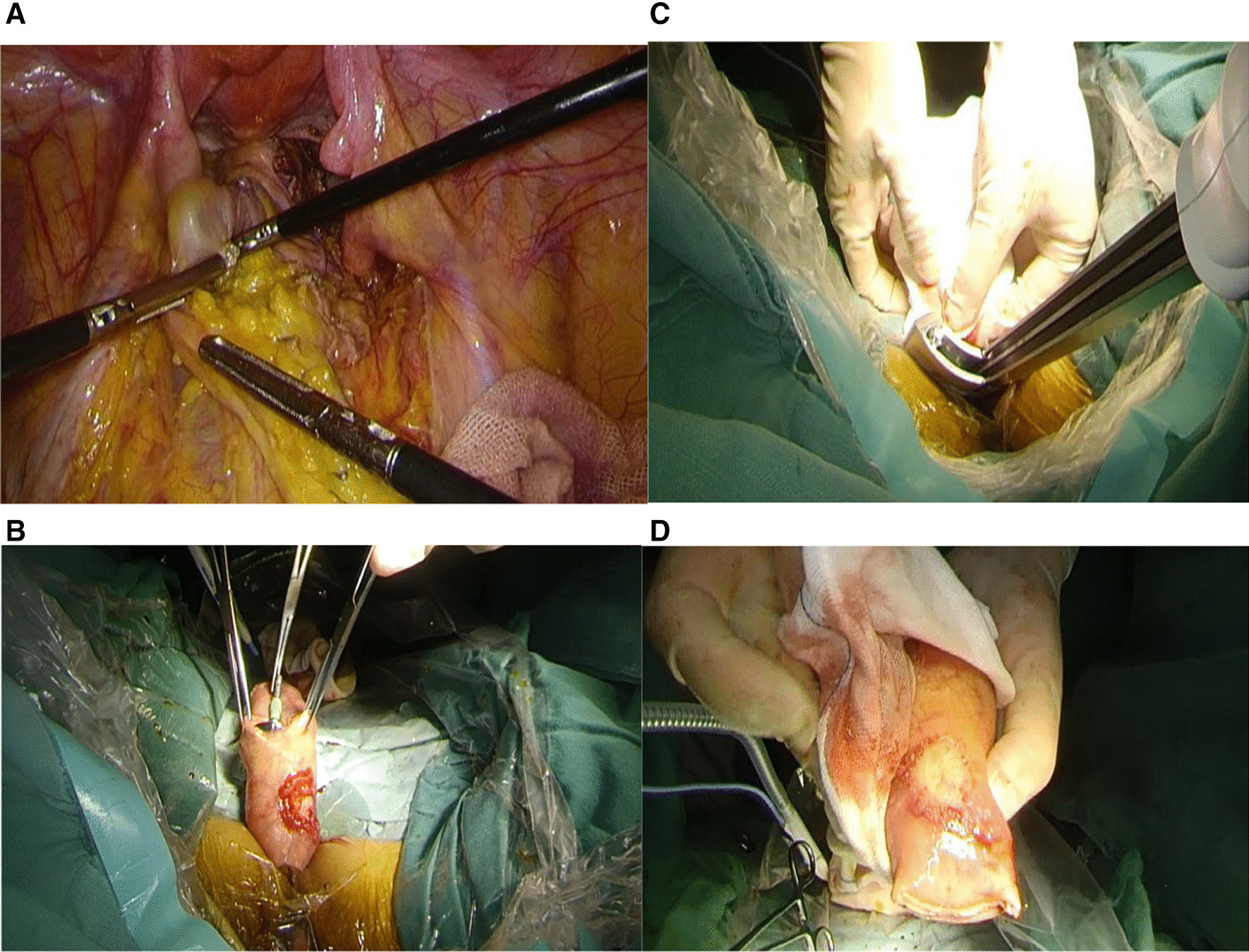
**A** Everted rectum with toothed ring forceps; **B** Rectal specimens pulled out of the body; **C** rectum and tumour were cut off under direct observation; **D** specimen after resection, check the cutting edge

**Fig. 3:**
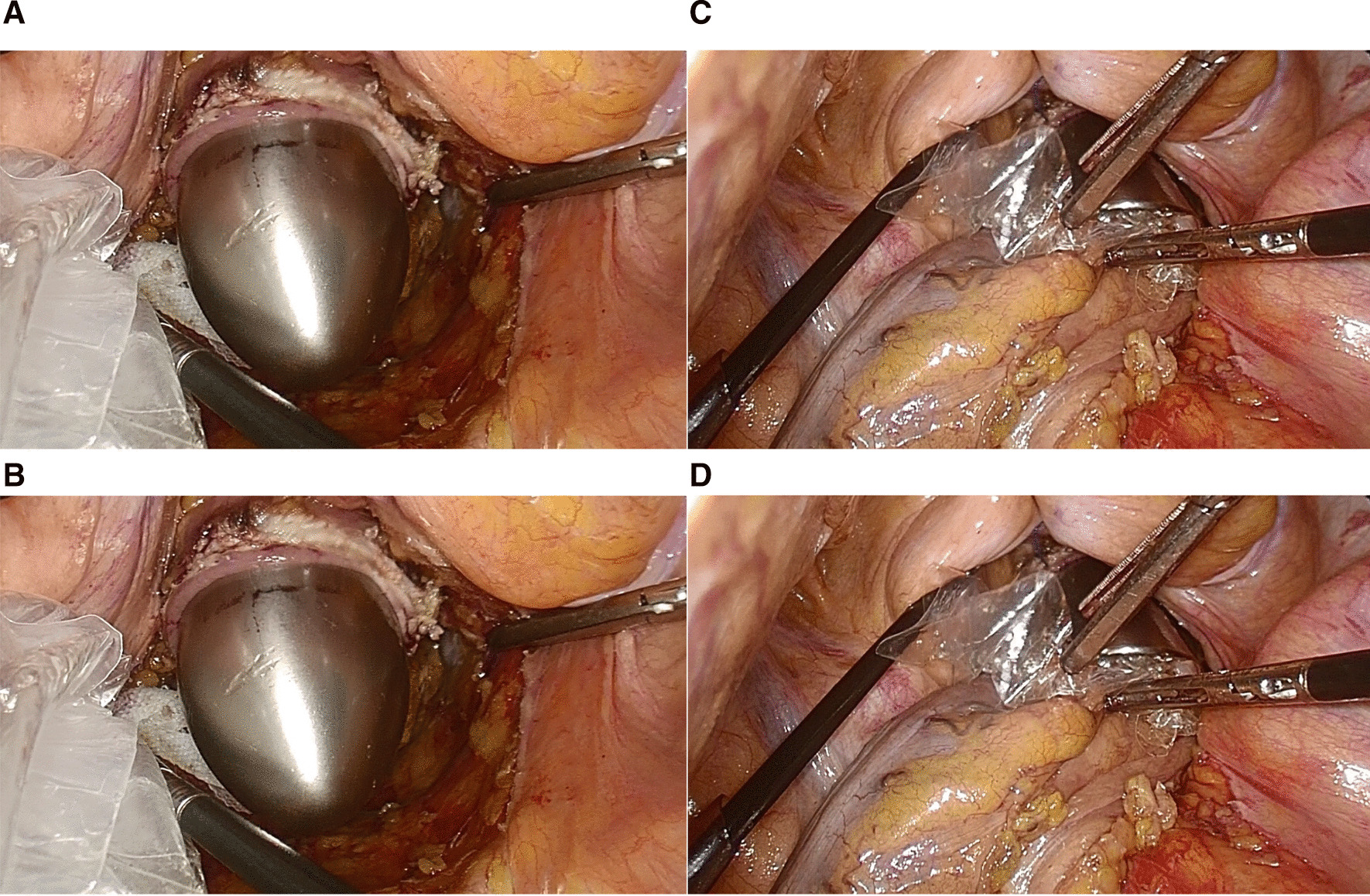
**A** Opening the distal rectum; **B** expansion of distal bowel with TEM instrument; **C** insert protective sleeve; **D** pulling out the specimen through the protective sleeve

(1) LAR-TAPT: after determining the position of the proximal sigmoid colon, the sigmoid mesentery was cut off, and the sigmoid colon was closed and cut off by a linear cutter stapler approximately 10 cm from the proximal end of the tumour. The next steps are to fully expand the anus, flush the intestinal cavity, extend the oval forceps through the anus, clamp and pull the closed end of the sigmoid colon, turn the sigmoid colon and rectum together with the tumour out of the body position. The intestinal cavity was rinsed and disinfected again with diluted povidone-iodine solution, the proximal intestinal tube of the tumour was opened, and the anvil head was placed into the abdominal cavity. The inferior margin of the tumour was accurately judged under direct observation, and the specimen was removed (Fig. 2). (2) HAR-TASE: the rectum was closed and cut off approximately 2 cm from the distal end of the rectal tumour by a linear cutter stapler, and the sigmoid colon was closed and cut off approximately 10 cm from the proximal end of the rectal tumour. A povidone-iodine ball was placed in the abdominal cavity around the closed end of the rectum. After proper isolation and protection, the closed end of the rectum is opened, and a sterile plastic protective sleeve or transanal endoscopic microsurgery (TEM) sleeve is placed. After the anvil head is inserted into the abdominal cavity through the protective sleeve, the oval forceps are extended through the anus to clamp the closed end of the distal end of the tumour. Pull the surgical specimen out of the body slowly. The incised rectal stump was closed again by a linear cutter stapler. The pelvic cavity was thoroughly washed with a large amount of diluted povidone-iodine solution (Fig. 3) [11].

#### Digestive tract reconstruction

**Fig. 4:**
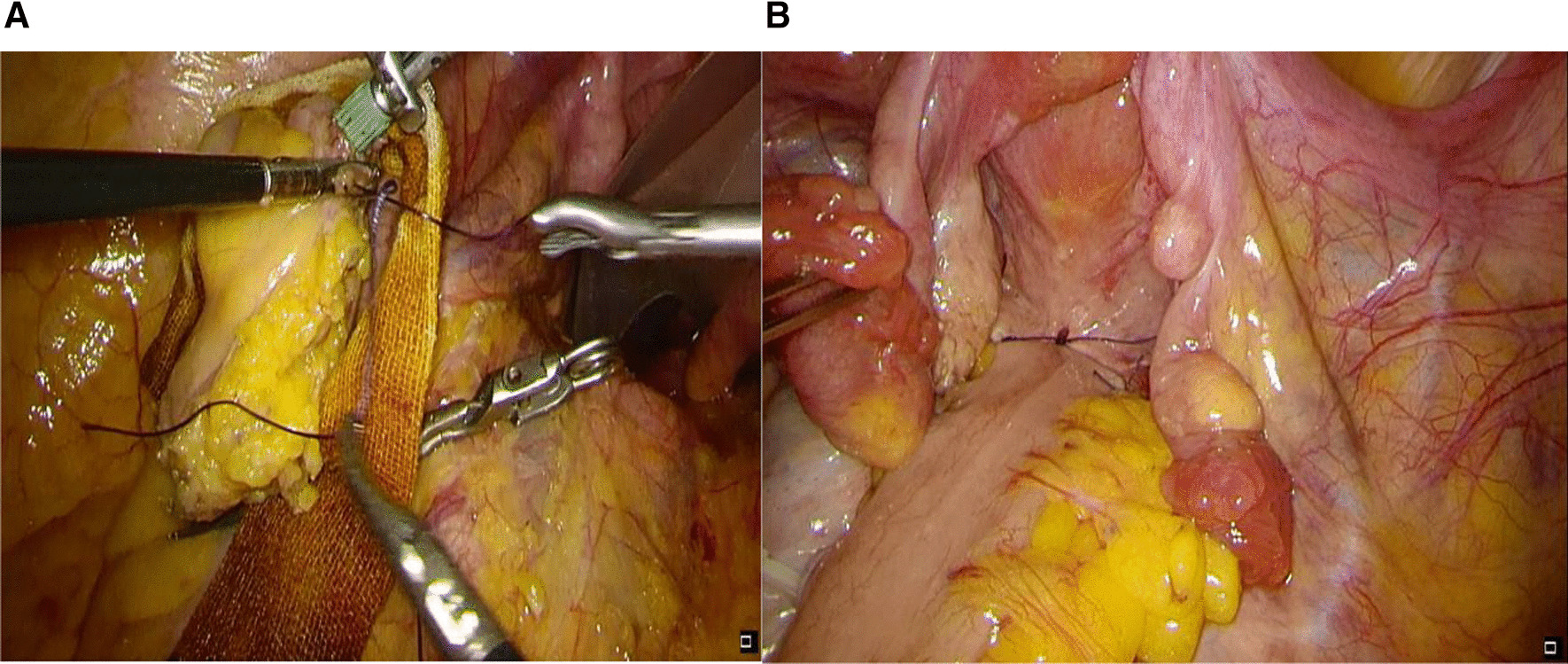
**A** Proximal purse string suture, iodophor gauze protection, proximal clipping; **B** complete reconstruction, pelvic floor reconstruction)

When the proximal sigmoid colon is closed, the purse string suture is manually sutured under endoscopy. Then, the closed end is removed, the intestinal cavity is opened, the anvil head is placed, and the purse is embedded. Under direct observation, it was end-to-end anastomosed with the central rod of the stapler inserted through the anus. After the intestinal cavity inflation test confirmed that the anastomosis was satisfactory, the pelvic drainage tube was placed, and the pelvic floor peritoneum was closed (Fig. 4) [11].

## Results

The average operation time was 304.36 ± 45.04 min, and the intraoperative bleeding was 76.31 ± 61.12 ml. Preventive ileostomy was performed in 7 patients, including 4 cases after neoadjuvant radiotherapy and chemotherapy, 2 cases of colon anal anastomosis and 1 case of long-term dialysis complicated with renal failure. The average total number of lymph nodes was 13.00 ± 6.66. The first time of getting out of bed was 14.42 ± 3.56 h, first anal exhaust was after 15.26 ± 8.92 h, and the time of fluid inflow was 2.94 ± 1.12 days. One patient developed pulmonary infection after the operation. The patient was complicated with COPD before the operation and improved after anti-infection treatment. Two patients had transient intestinal obstruction, which was relieved after 3–5 days of conservative treatment. No anastomotic leakage or pelvic abscess occurred in this group.

## Discussion

Since Franklin [12] reported the transanal pull-out technique after colonic segment resection in 1993, the NOSES technique has been continuously developing, and reports on transanal or vaginal pull-out specimens of colorectal benign and malignant tumours have been increasing. In 2006, Person [13] first reported the study of anal pull-out after total mesorectal resection for rectal cancer, which opened the prelude to the wide application of NOSES in colorectal cancer surgery. In 2017, the China NOSES Alliance reported a total of 718 cases of colorectal cancer specimens removed through the natural lumen in 79 hospitals [14], indicating that NOSES for colorectal cancer has been widely performed throughout the country. Since 2016, our centre has been performing laparoscopic colorectal cancer NOSES surgery. Currently, more than 100 cases have been completed, and good clinical results have been achieved.

Whether to preserve the left colonic artery in laparoscopic rectal cancer surgery has always been controversial, but an increasing number of studies have shown that preserving the left colonic artery can significantly increase the blood supply of the proximal intestinal canal of the anastomosis and reduce the risk of anastomotic leakage [15]. Moreover, on the premise of thoroughly cleaning the lymph nodes at the root of the inferior mesenteric artery, preserving the left colonic artery will not affect the radical effect of the tumour [16].

The authors believe that complete D3 lymph node dissection can also be achieved as long as the root of the inferior mesenteric artery and its branches are exposed by ossification (Fig. 1). In addition, the cases of the left colonic artery were retained. During the operation, the colour of the sigmoid colon at the proximal end of the anastomosis and blood leakage at the broken end were significantly improved. There was basically no need to worry about the blood supply of the intestine during the anastomosis, and the operation safety was higher. There was no anastomotic leakage in 19 patients.

There was no auxiliary incision in the abdomen. The most prominent advantages in the perioperative period are light abdominal wall pain, less analgesic demand, early postoperative activities and rapid recovery of gastrointestinal function [17]. The time of getting out of bed for the first time after operation was 14.42 ± 3.56 h, anal exhaust was 15.26 ± 8.92 h, and postoperative fluid intake time was 2.94 ± 1.12 days, which fully demonstrated the advantages of rapid recovery after NOSES [18].

Although NOSES has obvious advantages over traditional laparoscopy in short-term postoperative recovery and incision complications, there are still many disputes about this operation. The focus of attention is whether the principle of sterility and tumour-free status can be well-followed in specimen removal and digestive tract reconstruction without incision in the abdominal wall during NOSES operation.

Costantino et al. [19] conducted a comparative study on the problem of intraperitoneal contamination during NOSES and traditional laparoscopic surgery. The obtained results showed that the intraperitoneal bacterial contamination rates in the NOSES group and traditional laparoscopic group were 100% and 88%, respectively, with a significant difference; however, there was no significant difference between the two groups with clinical symptoms.

In view of the sterility problem, the author believes that it is necessary to open the intestinal cavity, pull out the specimen through the intestinal cavity, or place the stapler during NOSES, which does have the risk of abdominal cavity pollution, but this risk can be reduced or even avoided as much as possible through adequate preoperative intestinal preparation and targeted prevention during operation.

When the specimen was taken by cutting and pulling out, the distal intestinal cavity was first flushed with diluted iodophor solution, the broken end of the intestinal tube was protected with iodophor gauze, the broken end of the intestinal tube was opened under the condition of full disinfection protection, and then the endoscope protective sleeve was placed. The insertion of the nail base and the removal of the standard are performed in a protective sleeve (Fig. 3) to avoid the risk of aseptic contact between the nail holder and the intestinal cavity and the risk of contact between the tumour specimen and the tumour-free intestinal cavity. During digestive tract reconstruction, the purse should be sutured first, and then the proximal intestinal cavity should be opened to shorten the opening time of the intestinal cavity and reduce the chance of contact between the instrument and the intestinal cavity. Moreover, before opening the intestinal cavity, the free end of the intestinal canal should also be protected and isolated with iodophor gauze, and the proximal end of the free end should be clamped with pug pliers to block the descending of the contents of the intestinal cavity (Fig. 4). After the operation, the basin and abdominal cavity should be washed successively with a large amount of diluted iodophor solution and distilled water [11].

During LAR-TAP, the greatest controversy is whether the tumour specimen will spread retrogradely due to extrusion in the process of valgus pull-out. The author believes that after the mesentery is completely free, in just a few seconds after the specimen is pulled out, the tumour cells have to complete the process of abscission, blood entry, retrograde and reflux, and the risk of diffusion and metastasis is minimal. Of course, to further reduce the risk of tumour formation, it is very important to select appropriate cases, fully free the mesangium, and ensure the smooth and fast process of LAR-TAP [20]. Regarding the 5-year DFS or OS rate, previous studies [21–23] provided data on the 5-year DFS rate and OS rate. There was no significant difference in 5-year DFS between the La-NOSE and CL groups.

In 2017, Guan Xu et al. [14] reviewed and analysed the clinical data of 718 cases of colorectal tumour NOSE surgery in 79 hospitals. The obtained results showed that the overall incidence of postoperative complications was 10.6%, of which the incidence of abdominal infection was only 0.8%. Wang et al. believes that the aseptic risk of NOSE operation can be completely controlled as long as full preparation is made before operation and attention is given to operation details during surgery. To prevent the formation of new tumours, it is necessary to adapt the abovementioned procedure to the reasonable selection of the population and use standardized operation during the surgery [20]. Regarding 5-year DFS or OS rate, Shihao Wang’s meta-analysis included 16 studies comprising 2266 patients,showed that the number of lymph node dissection, 3-year disease-free and overall survival in the NOSES group were comparable with those in the CL group [24]. Meanwhile, for the standardization and safety of NOSE surgery, in 2019, the International Alliance of NOSES issued international consensus on natural orifice specimen extraction surgery (NOSES) for colorectal cancer [25]. In order to better develop NOSES technology globally, we should continue to improve NOSES technology based on this consensus, so as to benefit more patients.

## Conclusion

The short-term efficacy of 19 cases of rectal cancer NOSES in this group shows that laparoscopic anterior resection of rectal cancer with LCA preservation and NOSES is safe and feasible, sterile and tumour-free risk is controllable, and the short-term postoperative efficacy is satisfactory, especially in postoperative recovery and pain control. NOSES is worthy of further clinical promotion by experienced surgeons for appropriate patients.

## Data Availability

All data generated or analysed during this study are included in this published article.

## Abbreviations

NOSES: Natural orifice specimen extraction surgery
LCA: Left colonic artery
TME: Total mesorectal excision
PANP: Pelvic autonomic nerve preservation
LAR-TAPT: Transanal pull-through for low rectal cancer
HAR-TASE: Transanal specimen extraction for medium and high rectal cancer
IMA: Inferior mesenteric artery
SHP: Superior hypogastric plexus
IMV: Inferior mesenteric vein
TEM: Transanal endoscopic microsurgery
